# Stem cell treatment for multiple sclerosis

**DOI:** 10.1007/s00415-016-8284-z

**Published:** 2016-09-16

**Authors:** W. Owen Pickrell, Neil P. Robertson

**Affiliations:** 1 Neurology and Molecular Neuroscience, Swansea University Medical School, Swansea University, Swansea, SA2 8PP UK; 2 Institute of Psychological Medicine and Clinical Neurosciences, Cardiff University, Cardiff, CF14 4XW UK

Multiple sclerosis (MS) has long been considered an immune based disease and some of the earliest attempts to devise effective interventions were based on experience gained from the treatment of systemic immune disorders and included the use of less specific immunomodulating agents and myeloablative treatments including autologous hemopoietic stem cell transplantation (aHSCT). These either proved ineffective or were not widely adopted because of difficulties generating persuasive evidence for long-term benefit and unacceptably high morbidity and early mortality (in early studies, a mortality of 5 % or more with bone marrow transplantation). In particular for aHSCT it has proven difficult to generate informative control groups, blind treatment groups and to separate the effects of conditioning treatments prior to aHSCT and the aHSCT itself. Despite this some centres have continued to use aHSCT since 1995 in a small number of patients with severe advanced disease resistant to standard interventions. Because of these issues more targeted approaches to modulating the immune system to treat MS has generally been pursued over the last two decades. This has clearly been successful and continues to gain momentum so that a number of treatments are now available for different stages and levels of disease aggression.

However, in this intervening period there have also been parallel advances in aHSCT and in particular a reduction in treatment associated mortality and morbidity. In addition there has been an improved understanding of MS therapeutics with a trend towards earlier and more aggressive interventions and an evolution in clinical trial design. Recently, further pressure to re-examine the relevance of aHSCT in MS has also been provided by a flurry of high level media activity following the publication of a number of open label studies and questions concerning the suitability of this technology have become a common topic of discussion between patients and clinicians within specialist clinics.

aHSCT involves harvesting hemopoietic stem cells before completely ablating (immunoablation) or partially ablating the immune system with combinations of chemotherapy, monoclonal antibodies and anti-thymocyte globulin. The harvested stem cells are then reinfused, with or without subtype selection, to reconstitute the immune system. The process could be considered a partial or complete “reprogramming” of the immune system. In this month’s journal club we discuss three papers describing aHSCT for the treatment of MS. The first paper describes a case series of patients treated with nonmyeloablative aHSCT. The second and third papers describe results from phase 2 trials of myleoablative aHSCT.

## Association of nonmyeloablative hemopoietic stem cell transplantation with neurological disability in patients with relapsing-remitting multiple sclerosis

This paper describes a case series of 151 patients with MS treated at a single US hospital. 22 (15 %) patients were conditioned with cyclophosphamide and alemtuzumab, with the remainder [129 (85 %)] receiving cyclophosphamide and anti-thymocyte globulin before autologous transplantation of unselected hemopoietic stem cells. 123 (81 %) had relapsing-remitting and 28 (19 %) had secondary progressive MS. Fifty five patients were treated on the study protocol and met the following criteria: relapsing-remitting MS, Extended Disability Status Scale (EDSS) scores of between 2.0 and 6.0, received treatment with at least 1 FDA approved drug [mostly beta-interferon but also natalizumab (27 %) and fingolimod (8 %)], at least two corticosteroid treated relapses within the last year or one corticosteroid treated relapse and gadolinium enhancing lesions on MRI. Ninety six patients were treated off the study protocol on a compassionate basis for reasons including having secondary progressive disease, an EDSS score greater than 6.0 or particularly disabling disease. The patients had regular EDSS, Multiple Sclerosis Functional Composite (MSFC), Neurologic Rating Scale (NRS) and short form 36 quality of life assessments as well as MRI scans with gadolinium and were followed up for a median of 2 years.

There were no deaths during the treatment. 14 (9 %) patients had a post-transplant autoimmune adverse event [immune-mediated thrombocytopenia (ITP), hypothyroidism or hyperthyroidism] with the proportion being higher in the group receiving alemtuzumab compared with anti-thymocyte globulin (9 % compared with 4 %).

The primary end point was change in EDSS score. The mean EDSS score for the whole group improved significantly after transplant, with 50 % and 64 % of patients demonstrating an improvement in EDSS score of greater than 1.0 point at 2 and 4 years, respectively. NRS and MSFC scores also improved significantly after treatment. 80 % and 68 % of patients had no new relapses, gadolinium enhancing lesions on MRI or increase in their EDSS score (disease free survival) at 2 and 4 years, respectively. 89 % and 80 % of patients had no new relapses at 2 and 4 years, respectively. A subgroup analysis suggested no benefit in patients with progressive disease not having relapses before treatment.

*Comment*: This study was a case series and not a trial and contained a heterogeneous mix of patients with both relapsing and progressive disease (despite the title). A significant proportion of patients (63 %) were treated off study protocol on a compassionate basis and the majority of patients had not received standard high efficacy treatments. This together with the varying conditioning regime including the use of alemtuzumab exemplifies the difficulties in reaching firm conclusions on the effect of aHSCT. On a positive note, there were no deaths and a relatively low proportion of treatment related complications when compared to other studies, which is likely to be due to the use of a nonmyeloablative conditioning regime. EDSS scores were presented as whole group averages, however, there was a significant improvement in EDSS score for the majority of patients and 80 % of patients achieved disease free survival at 2 years.

Burt RK et al. (2015) JAMA 313(3):275–284.

## High-dose immunosuppressive therapy and autologous hemopoietic cell transplantation for relapsing-remitting multiple sclerosis (HALT-MS)

This paper reports an interim analysis of the HALT-MS trial. HALT-MS is a single arm, multicentre, phase 2 trial of immunoablation (using carmustine, etoposide, cytarabine, melphalan and anti-thymocyte globulin) followed by autologous transplantation of CD34-selected stem cells. Twenty five patients were treated who had: relapsing-remitting MS, an EDSS score of between 3.0 and 5.5, and 2 or more clinical relapses in 18 months despite being on treatment. The patients had a median EDSS score of 4.5 and had failed a median of 3 treatments at inclusion. Follow-up included regular EDSS, MSFC and 29-item Multiple Sclerosis Impact Scale assessments as well as regular MRI scans with gadolinium. The median length of follow-up was 3.5 years.

One patient had a pulmonary embolus during the stem cell mobilisation phase and did not proceed further with the study. One patient died 2.5 years after treatment due to MS progression and another patient died 3.5 years after treatment due to worsening of asthma. Both of these patients had met the end point of the trial before death having had neurological progression and a relapse in the context of aseptic meningitis, respectively. The authors report 130 grade 3 (severe) and 94 grade 4 (life-threatening or disabling) toxic events, most of which were expected cytopaenias or infections.

The primary end point was the time to treatment failure. At 2 and 3 years, the overall event-free survivals (no progression in EDSS, clinical relapses or new gadolinium enhancing lesions on MRI) probability, were 83 % and 78 % respectively. The EDSS score had improved from baseline by a median of 0.5 points after 3 years.

*Comment:* These were the preliminary results from a small, single-arm trial. The authors give detailed information about the frequency of severe and life-threatening adverse effects that can be expected following complete immune system ablation. However, the two deaths described did not seem to be directly related to treatment. The event-free survival probabilities are impressive but comparable to previous phase II and III studies of high efficacy treatments already available for relapsing disease.

Nash RA et al. (2015) JAMA Neurol 72(2):159–169.

## Immunoablation and autologous hemopoietic stem cell transplantation for aggressive multiple sclerosis: a multicenter single-group phase 2 trial

This was a single arm, multicentre, phase 2 trial of immunoablation (using cyclophosphamide, busulfan and rabbit anti-thymocyte globulin) followed by autologous transplantation of CD34-selected stem cells, across three Canadian hospitals. Twenty four patients with aggressive MS were included, 50 % had relapsing-remitting and 50 % had secondary progressive disease. All patients had multiple early relapses, an EDSS score of at least 3.0 within 5 years of diagnosis and evidence of ongoing clinical disease activity despite at least 1 year of immunosuppressive treatment (mostly beta-interferon). The patients had regular EDSS and MSFC assessments as well as MRI scans with gadolinium and were followed up for a median of 6.7 years.

One patient died of hepatic necrosis and sepsis 62 days after transplantation and another patient required intensive care admission before recovering fully. All 23 surviving patients were free of clinical relapses and new gadolinium enhancing lesions for the duration of follow-up (median of 6.7 years). 17 (70 %) patients had no further progression in their EDSS scores after treatment and 8 (35 %) patients had a sustained improvement in their EDSS scores 3 years after treatment. Rates of brain atrophy were not significantly different to those of healthy volunteers.

*Comment*: Although this was a small, single-arm trial, a broad range of outcome measures including measures of brain atrophy have produced some persuasive results over a relatively long period of follow-up. All participants who completed the trial had cessation of clinical relapses and no new lesions on MRI. A significant proportion of participants had a functional improvement 3 years after treatment, despite a high baseline level of disability and half the participants having secondary progressive disease. The level of treatment success in this study may have been due to the relatively high doses of immunoblative chemotherapy used and the selection of CD34 cells for transplantation. However, the results need to be balanced by the significant toxicity including a 4 % mortality rate.

Atkins HL et al. (2016) Lancet 388(10044):576–585.

Conclusion: Overall, the results of these studies offer some encouragement for continuing investment in the role of aHSCT in MS. However, there is an undeniably high incidence of severe adverse events, including death, that needs to balance against any benefit which must be sustained over the longer term so that further follow-up will be essential. In addition appropriate patient selection will be key and it now seems clear that aHSCT is not effective in progressive patients and the ethics of including this group in future trials seem questionable. None of these studies shed further light on the relative effect of conditioning versus transplantation, which must be resolved before wider use is considered. Finally, it would also be of value to compare the efficacy of aHSCT directly against currently available high efficacy treatments despite the difficulties in trial design this would generate.

